# Transcriptomics Analysis of Circular RNAs Differentially Expressed in Apoptotic HeLa Cells

**DOI:** 10.3389/fgene.2019.00176

**Published:** 2019-03-13

**Authors:** Bilge Yaylak, Ipek Erdogan, Bunyamin Akgul

**Affiliations:** Non-Coding RNA Laboratory, Department of Molecular Biology and Genetics, Izmir Institute of Technology, Izmir, Turkey

**Keywords:** apoptosis, circular RNA, RNA-seq, transcriptomics, HeLa

## Abstract

Apoptosis is a form of regulated cell death that plays a critical role in survival and developmental homeostasis. There are numerous reports on regulation of apoptosis by protein-coding genes as well as small non-coding RNAs, such as microRNAs. However, there is no comprehensive investigation of circular RNAs (circRNA) that are differentially expressed under apoptotic conditions. We have performed a transcriptomics study in which we first triggered apoptosis in HeLa cells through treatment with four different agents, namely cisplatin, doxorubicin, TNF-α and anti-Fas mAb. Total RNAs isolated from control as well as treated cells were treated with RNAse R to eliminate the linear RNAs. The remaining RNAs were then subjected to deep-sequencing to identify differentially expressed circRNAs. Interestingly, some of the dys-regulated circRNAs were found to originate from protein-coding genes well-documented to regulate apoptosis. A number of candidate circRNAs were validated with qPCR with or without RNAse R treatment as well. We then took advantage of bioinformatics tools to investigate the coding potential of differentially expressed RNAs. Additionally, we examined the candidate circRNAs for the putative miRNA-binding sites and their putative target mRNAs. Our analyses point to a potential for circRNA-mediated sponging of miRNAs known to regulate apoptosis. In conclusion, this is the first transcriptomics study that provides a complete circRNA profile of apoptotic cells that might shed light onto the potential role of circRNAs in apoptosis.

## Introduction

Apoptosis is a tightly regulated mechanism of type 1 programmed cell death that mediates balance between survival and cell death. Apoptosis mainly proceeds with the extrinsic death receptor pathway or the intrinsic mitochondrial dysfunction pathway (Vince and Silke, 2009; Fuchs and Steller, 2011). Functional apoptosis pathway is vital for tissue homeostasis and organ development. Accordingly, dysregulation of apoptosis is associated with a wide range of diseases such as cancer, autoimmune diseases, neurodegenerative diseases, and acute pathologies (Favaloro et al., 2012). Therefore, much effort has been made to unravel molecular pathways that trigger and regulate apoptosis.

During past decades, a plethora of studies revealed that considerable amount of DNA called “dark matter of genome is transcribed but not translated” (Bertone et al., 2004). MicroRNAs (miRNAs) and long non-coding RNAs (lncRNAs) are well-known examples of non-coding RNAs (ncRNAs) (Lewis et al., 2005; Lander, 2011). Recent studies revealed yet another type of ncRNAs, circRNAs, as a novel type of endogenous ncRNAs that has the potential to regulate not only the protein-coding genes but also ncRNAs as well (Salzman et al., 2013; Jeck et al., 2013; Memczak et al., 2013; Huang S. et al., 2017).

Circular RNAs are typically generated by back-splicing of exons from pre-mRNAs with a 3’-to-5’ phosphodiester bond at the junction site (Chen, 2016). Since they do not possess a 5’ cap or a poly(A) tail, they are frequently missed in poly(A)^+^ RNA profiling studies. Also, the unusual back-splicing generates a read that has been historically filtered out during conventional RNA-seq studies. The recent development of non-polyadenylated sequencing technology followed by exonuclease-mediated degradation of linear RNAs by RNAse R resulted in the discovery of thousands of circular RNAs widely expressed in human (Suzuki et al., 2006). The majority of circular RNAs are conserved across species, and generally reveal tissue/developmental-stage-specific expression (Qu et al., 2015). Searching for out-of-order arrangement of exons known as a “backsplice” in combination with computational pipelines facilitated the genomewide identification of circRNAs (Jeck and Sharpless, 2014). Non-etheless, identification of backsplicing reads can be challenging because of high false positive rates of computational tools. These apparent backsplicing sequences can originate from different mechanisms such as tandem duplication, RNA-trans splicing and reverse transcriptase template switching rather than exonic circular RNAs (Cocquet et al., 2006; McManus et al., 2010; Jeck and Sharpless, 2014).

Circular RNAs can regulate gene expression through various mechanisms. Perhaps, one of the most prominent functions of circRNAs is to modulate endogenous miRNA activities by acting as miRNA sponges (Hansen et al., 2013; Memczak et al., 2013). CircRNAs might also regulate transcription by interacting with RNA polymerase II and U1 snRNP or various RNA binding proteins such as Mbl (Ashwal-Fluss et al., 2014). Moreover, the canonical pre-mRNA splicing and circularization may compete with each other, resulting in a decrease in the amount of the linear mRNA (Ashwal-Fluss et al., 2014). Besides, if the translation start site of an mRNA is included in the circularized exon, the remaining portion of the mRNA will miss a proper translation initiation site, negatively affecting its translation (Huang G. et al., 2017). Although circular RNAs were initially categorized as non-coding RNA, it was obvious that a portion of circRNAs held the potential for translation, especially those that retain an intact open reading frame. Spectacularly, cap-independent translation of a subset of circular RNAs has been reported recently, paving the way for truncated translation products from circRNAs (Pamudurti et al., 2017).

There are only a few studies that report the involvement of circRNAs in apoptosis. Circ_016423 has been reported to have a high possibility of regulating apoptosis in ischemic prenumbral cortex, potentially through harboring binding sites for various miRNAs (Mehta et al., 2017). Hsa_circ_0010729 was reported to regulate proliferation and migration as well as apoptosis in hypoxia-induced human umbilical vein endothelial cells (HUVECs) by targeting miR-186-HIF-alpha axis (Dang et al., 2017). MFACR possesses a binding site for miR-652-3p, which in turn regulates the MTP18 mRNA, which codes for a nuclear-encoded mitochondrial membrane protein associated with apoptosis in cardiomyocytes (Wang et al., 2017).

Holdt et al. (2016) reported that overexpression of circANRIL, the circular form of lncRNA ANRIL, induces apoptosis in HEK-293 cells. Further, circular Rar1, circRar1, was shown to regulate apoptosis in N2a mouse neuroblastoma cells (Nan et al., 2016). Pro-apoptotic lncRpa and apoptosis-related circRar1 were shown to be regulated by miR-671. However, a complete genomewide profile of dysregulated circular RNAs under apoptotic conditions remains unknown. Here we report the first genome-wide profile of differentially expressed circular RNAs in apoptotic HeLa cells and provide bioinformatics data that suggest the potential translatability of certain candidate circRNAs.

## Materials and Methods

### Cell Culture, Drug Treatment and Measurement of Apoptosis

HeLa cells were obtained from DKFZ GmbH (Germany). Cells were cultured in RPMI 1640 (with L-Glutamine, Gibco, United States) supplemented with 10% heat-inactivated fetal bovine serum (FBS) (Gibco, United States) and 1% penicillin-streptomycin (Gibco) in an atmosphere of 5% CO_2_ at 37°C. Based on the initial dose and time kinetics, the subsequent treatments were carried out in the following conditions (1) cisplatin (Santa Cruz, United States) at a concentration of 80 μM for 16 h; (2) doxorubicin (Cell Signaling, United States) at a concentration of 0.5 μM for 4 h; (3) anti-Fas mAb (Millipore, United States) at a concentration of 0.5 μg/ml for 16 h; and (4) TNF-α (Millipore, United States) at a final concentration of 125 ng/ml TNF-α with 10 μg/ml cycloheximide (Applichem, Germany) for 8 h. Untreated and dimethylsulfoxide (DMSO)-treated (0.1%) cells were used as negative control.

Following three biological replicates of drug treatments, 0.5 × 10^6^ cells were trypsinized by 1× Trypsin-EDTA (Gibco, United States) and washed in 1X cold PBS (Gibco, United States), followed by resuspension in 1× Annexin binding buffer (Becton Dickinson, United States). The resuspended cells were stained with Annexin V-PE (Becton Dickinson, United States) and 7AAD (Becton Dickinson, United States) for 15 min in dark at room temperature and analyzed by FACS Canto (Becton Dickinson, United States).

Control and apoptotic cells were also stained with NucRed^TM^ Dead 647 ReadyProbes^TM^ Reagent (Invitrogen) to visualize apoptotic cells under fluorescence microscopy. HeLa cells (0.4 × 10^6^ cells/well) were seeded in a six-well plate and treated with 4 drugs as explained above. DMSO (5%) (v/v) was used as positive control. At the end of treatment period, 2 drops/ml of medium was applied on the cells and cells were monitored by a fluorescent microscope (Zeiss Observer Z1) after 30 min of incubation in dark.

### CircRNA Deep-Sequencing and Bioinformatics Analyses

Control and treated cells were sent to NOVAGENE (Hong Kong) in RNAlater for RNA isolation and deep sequencing using a previously published method optimized for circular RNA identification (Szabo and Salzman, 2016). Three biological replicates of total RNAs from control and treated cells were then subjected to poly(A) tail elimination, rRNA depletion and RNase-R treatment for circRNA enrichment prior to deep sequencing (Szabo and Salzman, 2016). The original raw data obtained from high throughput sequencing (Illumina HiSeqTM2500) were first transformed to Sequenced Reads containing read sequence and corresponding base quality (in FASTQ format) through Base Calling. Novel circRNAs were identified and annotated using CIRCexplorer (Zhang et al., 2014), which remapped the unmapped reads to the genome by tophat’s fusion-search to find back-spliced reads. The normalized expression of circRNAs in each sample was calculated based on the transcript per million (TPM) method (Zhou et al., 2010) using DESeq2 version 1.6.3 (Love et al., 2014) where the threshold was set as padj <0.05.

Log2 ratios were clustered by K-means and self-organizing maps (SOM) algorithms as well as TPM. CircRNA and miRNA pairing were annotated by miRSystem (Lu et al., 2012) for further analysis. All differentially expressed circular RNAs, origin mRNAs and potential miRNA binding sites were classified based on their fold change, pathway-drug specificity and source genes. Translation potential of circRNAs was examined by CircInteractome ^1^, Ensembl ^2^, NCBI ORF Finder ^3^, IRESitefootnoteiresite.org/, and SRAMP: prediction of mammalian N6-methyladenosine (m^6^A) sites ^4^.

Initially, we focused on cisplatin-treated samples due to the widespread use of cisplatin in cancer treatment. Thus, 109 differentially expressed circular RNAs in cisplatin-treated samples were subjected to further bioinformatics analyses. StarBase ^5^ was used to investigate potential miRNA-circRNA interactions. miRNAs with potential binding sites were then fed into miRSystem ^6^ to identify putative target mRNAs as well as pathways that might be affected by the potential miRNA-circRNA interactions. To examine the translation potential of circRNA candidates, exon sources of circular candidates were downloaded from Ensembl (see text footnote 2). Those that originate from the first exons were searched for the presence of a potential internal ribosome entry site using IRESite (see text footnote 4). SRAMP (see text footnote 5) was used to analyze the presence of m6A sites on hsa_circ_0029693.

### Validation of circRNAs by Cloning

Total RNA was isolated from cisplatin (80 μM) and DMSO (0.1%) (Applichem, Germany) treated cells using TRIzol (Thermo Fisher Scientific, United States) according to the manufacturer’s instructions. For all candidates, divergent primers were designed by using the CircInteractome web tool (see text footnote 1). These primers were specific to amplify backsplice junctions, excluding the potential for the amplification of linear mRNAs (Table 1). Total RNAs were then treated with RNAse R (Epicenter) according to manufacturer’s instructions to eliminate the linear RNAs and to enrich the circRNAs. Briefly, 2 μg of total RNAs, 1 μl buffer and 5 units of RNase R were mixed in a final volume of 10 μl and incubated for 30 min at 37°C. Subsequently, RNA clean-up was performed with Nucleospin RNA kit (Macherey Nagel, Germany) according to the manufacturer’s instructions.

**Table 1:** List of divergent primers.

	Forward primer	Reverse primer
hsa_circ_0012992^b^	AGGCTTGGATCTTC AGCCATT	AGGTGGATGCACA AAATCTCAG
circ-HIPK3^b^	TATGTTGGTGGATCC TGTTCGGCA	TGGTGGGTAGACCA AGACTTGTGA
Beta-actin^a^	CTGGGACGACATGGAGAAAA	AAGGAAGGCTGGAA GAGTGC
hsa_circ_0014824^b^	ATCACAGGACCCA AACCTCTG	CTTGCGCTCTGGTG CAGAAT
hsa_circ_0029693^b^	TGCTAATGAATCG GGCACCT	TCTTTCCTTCCAT TTTTGTAGTTCC
hsa_circ_0029693^c^	AAGCCTTGAGGG AAATCAGA	TGGCCTCATTG TTAGTCCAG
GAPDH^a^	ACTCCTCCACC TTTGACGC	GCTGTAGCCAAATT CGTTGTC


*^*a*^Convergent primer, ^*b*^for qPCR amplification, ^*c*^for cloning.*

cDNAs were prepared from RNAse R^+^ and RNAseR^-^ RNA samples with ProtoScript^®^ first strand cDNA synthesis kit (NEB, United States) with random primers in a total volume of 50 ul according to the manufacturer’s instructions. The cDNA reactions contained 200 ng of total RNAs or equivalent of RNAse-treated samples. The candidate circRNAs were amplified by PCR using the following conditions: Initial denaturation at 95°C for 30 s, 35 cycles of denaturation at 95°C for 30 s, annealing at 60°C for 1 min, extension at 68°C for 1 min, 1 cycle of final extension at 68°C for 5 min. PCR products were run on 1% agarose gel to examine the size and purity of the amplified products. Bands of the correct size were extracted from the gel using the gel extraction kit (Macherey Nagel, Germany). The purified fragments were cloned into the pCR^®^II TA vector using the TA cloning kit (Thermo Fisher Scientific, United States) and sequenced at the BIOMER center (IZTECH, Turkey) to validate the backsplice junction of target circRNAs.

### qPCR Analyses

cDNAs prepared from RNAse R^+^ and RNAseR^-^ RNA samples were used as templates for qPCR with GoTaq q-PCR Master Mix (Promega, United States). Two step PCR amplification conditions were as follows: initial denaturation at 95 °C for 2 min, (40 cycles) denaturation at 95°C for 15 s and annealing/extension at 60°C for 1 min.

#### Statistical Analyses

All experiments were carried out at least in three biological replicates. Student’s *t* test was used to analyze the data. A *P*-value of 0.05 was considered statistically significant unless indicated otherwise.

## Results and Discussion

### Induction of Apoptosis and Deep Sequencing Analyses

**FIGURE 1:**
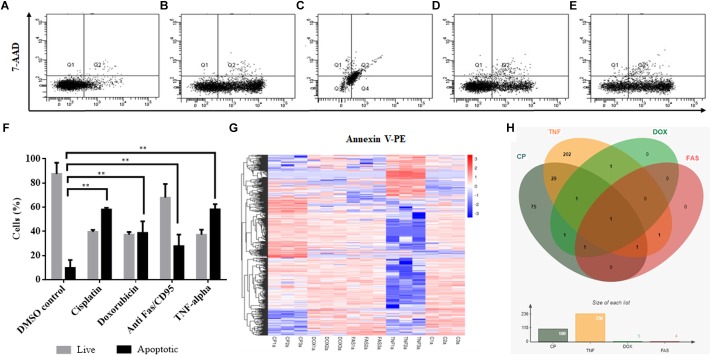
Induction of apoptosis and TPM cluster analysis of circRNAs in apoptotic HeLa cells. HeLa cells (0.5 × 10^6^) were treated with 0.1% DMSO as a control group for 16 h **(A)**, 80 μM cisplatin for 16 h **(B)**, 0.5 μM doxorubicin for 4 h **(C)**, 0.5 μg/ml anti-Fas mAb for 16 h **(D)** and 125 ng/ml TNF-α for 8 h **(E)**. Cells were then stained with Annexin V-PE (Becton Dickinson, United States) and 7AAD (Becton Dickinson, United States) and analyzed by FACS Canto (Becton Dickinson, United States). Annexin V-positive cells were regarded as early apoptotic cells **(F)**. **(G)** The overall TPM cluster analysis. The normalized expression of circRNAs in each sample is calculated based on the TPM method (Zhou et al., 2010). The normalized circRNA expression levels are clustered based on log10(TPM+1) value where the red color represents circRNAs with high expression level while the blue color represents circRNAs with low expression level. The color from red to blue represents the log10(TPM+1) value from large to small. 1a, 2a, and 3a refer to each of the three biological replicates (C is control DMSO). **(H)** Distribution of differentially expressed circRNAs in apoptotic HeLa cells. CP, cisplatin; FAS, anti-Fas mAb; TNF, TNF-α, and DOX, doxorubicin. All experiments were performed in triplicates. *p* < 0.005.

Cisplatin and doxorubicin treatment were performed to induce apoptosis by intrinsic pathway while anti-Fas mAb and TNF-α treatments were carried out to trigger the extrinsic pathway (Boatright and Salvesen, 2003; Morgan et al., 2013; Huang et al., 2018). In order to attain an apoptosis rate of approximately 50%, drug /ligand concentrations and incubation periods were optimized through dose and time kinetics (data not shown). Figures 1A–E shows the dot blot analyses of cell populations after treatment at optimum doses and times. All drug and ligand treatments caused cells to shift to the Annexin V+/7AAD– region (Figures 1B,D,E) except doxorubicin (Figure 1C), which caused cells to be included in Annexin V+/7AAD+ population. Apoptosis rates ranged between 27 and 58% (Figure 1F), depending on the treatment agent. Cisplatin and TNF-α caused the highest apoptotic rate (58%) while anti-Fas-treated population displayed a lower rate of apoptosis (27%) due to the desensitization of HeLa cells to FasR stimulation (Holmström et al., 1999). Control and apoptotic cells were also stained with NucRed^TM^ Dead 647 as a qualitative visualization of cell death (Supplementary Figure S1). The Annexin V+/7AAD– state of the cells were interpreted as cells being caught at the early apoptotic phase and thus they were deemed suitable for downstream steps.

To identify differentially expressed circRNAs, total RNAs were subjected to circRNA enrichment as explained in “Materials and Methods.” The number of raw reads ranged from 81,360,972 to 106,246,444 whereas clean reads ranged from 79,147,032 to 103,277,920 with an error rate of 0.02 to 0.03%. Q30 score was between 91 and 94.43%, indicating fairly a low error rate. The lowest percentage of total mapped reads was 68.77% whereas the highest percentage of total mapped reads was 94.3%. For all samples, the primary source of circRNAs was introns while getting reads from exons and intergenic sequences as well. TPM cluster analysis was conducted to obtain normalized circRNA expression data. Read count value from expression level analysis of circRNA was used as input data for K-means and SOM clustering (Supplementary Figure S2). This clustering revealed that circRNAs within the same cluster exhibited the same changing trend of expression levels under different conditions. Based on the TPM cluster analysis, it is apparent that circRNA expression differs among control, drug- and ligand-treated groups (Figure 1G). TNF-α and cisplatin treatment groups exhibited the highest number of dys-regulated circRNAs. However, the number of circRNAs differentially expressed in doxorubicin and anti-Fas mAb-treated HeLa cells were relatively low (Figure 1H). Venn diagram and size graph show that 109 circular RNA were differentially expressed in cisplatin-treated HeLa cells. Likewise a total of 236 circular RNAs showed differential expression in the TNF-α treated HeLa cells. Five and four circular RNA were differentially expressed in doxorubicin and anti-Fas mAb-treated HeLa cells, respectively. Consistent raw read numbers in three biological replicates of doxorubicin and anti-Fas mAb-treated samples suggest a drug-specific response rather than a technical problem, which begs for further investigation.

### Candidate Selection

Because the drugs and ligands used in our studies are potent inducers of not only apoptosis but also other cellular phenotypes such as stress, proliferation or cell cycle, we applied a number of filtering criteria to select candidate circRNAs for further analyses. Basically we selected the dys-regulated circRNAs that (1) had the highest log change difference between control and drug treated cells, (2) had a statistical significance of differential expression, and (3) originate from mRNAs that were reported or have the potential to regulate apoptosis. Based on these criteria, we selected 10 candidates that met our criteria the most. Among these candidates, hsa_circ_0099768, hsa_circ_0083543, hsa_circ_0043795, hsa_circ_0051666, and hsa_circ_0014824 were upregulated under apoptotic conditions whereas the rest of them were downregulated (Table 2). All candidates exhibited differential expression under cisplatin treatment whereas hsa_circ_0099768 was differentially expressed under all treatment conditions. hsa_circ_0083543 was the other candidate differentially expressed in cisplatin-, doxorubicin-, and anti-Fas-treated HeLa cells.

**Table 2:** Circular RNA candidates differentially expressed in apoptotic HeLa cells.

Candidates	*P*-Value	Log change	Parental mRNA
hsa_circ_0099768^∗^	2,29E-04	71.045	FGF14
hsa_circ_0083543^∗∗^	0.00053703	50.376	XPO7
hsa_circ_0043795	0.00068894	49.662	STAT5A
hsa_circ_0051666	0.0004384	50.975	BBC3
hsa_circ_0014824	9,18E-01	55.021	ARHGEF11
hsa_circ_0029693	0.00038011	–36.338	LATS2
hsa_circ_0081300	0.00039188	–36.291	TRRAP
hsa_circ_0012992	0.00014162	–53.499	USP33
hsa_circ_0080853	0.00011961	–53.925	PTPN12
hsa_circ_0097866	0.0001054	–54.228	TMEM132C


*Circular RNAs that were significantly down- and up-regulated upon treatment with all agents is listed below with their parental coding mRNAs. The majority of top 5 upregulated and downregulated circular RNAs originated from protein coding genes well-documented to regulate apoptosis. *P-*value, log change and apoptotic regulator function of the origin linear mRNA were considered as criteria in candidate selection. ^∗^ denotes the candidate common in all drug/ligand groups, ^∗∗^ denotes the candidate common in cisplatin, doxorubicin and anti-Fas group.*

### RNAse R Treatment and qPCR Validation

**FIGURE 2:**
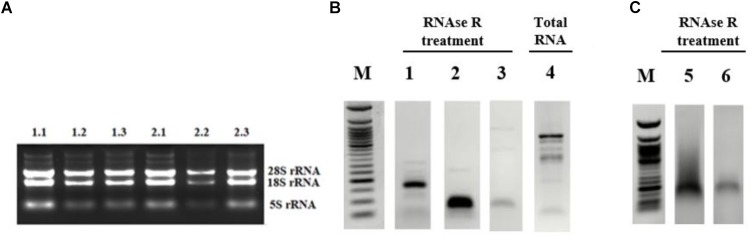
Validation of candidate circRNA by cloning. HeLa cells were treated with cisplatin in three biological replicates as explained in Materials and Methods. Total RNAs from each replicate of control DMSO (1.1–1.3) and cisplatin (2.1–2.3) were run on 1% agarose gel for visual analyses **(A)**. Total RNAs were treated with RNAse R to eliminate linear RNAs followed by reverse transcription and PCR amplification. Lanes 1, 2, 5, and 6 **(B,C)** are the PCR products from hsa_circ_0012992, hsa_circ_HIPK3, hsa_circ_0014824, and hsa_circ_0029693, respectively. The efficiency of RNAse R treatment was measured by PCR-amplifying the linear beta-actin mRNA before (Lane 4) and after (Lane 3) the RNAse treatment. The gel-purified PCR fragments were cloned into the pCR^®^II TA vector (Thermo Fisher Scientific, Unite States) and sequenced to identify the backsplice junction.

Although the bioinformatics algorithms eliminate artifacts from the circRNAs, additional experimental analyses are required to call the RNA-seq reads as circRNAs. Typically, RNAse R treatment is used to eliminate linear RNAs to prevent the potential amplification of tandem duplications (Panda et al., 2017). Additionally, the amplified products are sequenced to conclusively state the presence of a circRNA. To this extent, we used the total RNAs from control and cisplatin-treated cells (Figure 2A) and chose circ-HIPK3 as a positive control to test circRNA validation. Li et al. (2017) reported that circ-HIPK3 is indeed expressed in HeLa cells. We designed divergent primers using the Circinteractome tool (Dudekula et al., 2016) to amplify the positive control circ-HIPK3. qPCR was then performed with cDNAs that were reverse transcribed from both RNAse R^+^ and RNAse R^-^ samples.

First, we examined the efficiency of RNAse R treatment to eliminate linear RNAs using DMSO-treated control RNAs. As shown in Figure 2B, RNAse R treatment resulted in the loss of the linear beta actin mRNA (Lane 3 vs. 4). However, the positive control circ-HIPK3 was resistant to the treatment with RNAse R (Figure 2B, Lane 2). We also validated the resistance of our candidates, hsa_circ_0012992, hsa_circ_0014824, and hsa_circ_0029693 to RNAse R (Figure 2B, Lanes 1 and 2C). All these analyses showed that we were able to eliminate the linear RNAs and enrich the circular forms under our experimental conditions.

**FIGURE 3:**
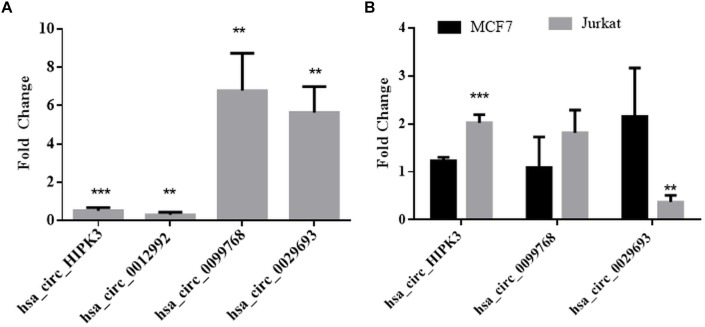
qPCR analyses of candidate circRNA expression. Cells were treated with DMSO (control) and cisplatin as explained in Materials and Methods. RNAse R-treated RNA samples were used to prepare cDNAs using ProtoScript^®^ first strand cDNA synthesis kit (NEB, United States) with random primers. GoTaq q-PCR Master Mix (Promega, United States) was used for qPCR analyses. Data collected in three biological replicates were normalized against DMSO control and GAPDH. ^∗∗^ and ^∗∗∗^ denote *p* ≤ 0.01 and *p* ≤ 0.001, respectively. **(A)** HeLa cells; **(B)** MCF and Jurkat cells.

We then performed qPCR analyses with total RNAs to quantitatively measure the circRNA transcripts in cisplatin-treated cells to validate circRNA-seq data. To this extent, we subjected three candidates, hsa_circ_0012992, hsa_circ_0099768, and hsa_circ_0029693 to qPCR analyses, which were shown to exist in the circular form (Figure 3). As shown in Figure 3, hsa_circ_0012992 and hsa_circ_HIPK3 were down-regulated 3.3- and 2-fold, respectively, while hsa_circ_0099768 and hsa_circ_0029693 were upregulated 6.7- and 5.6-fold, respectively. We then carried out cisplatin treatments in two other cell lines, Jurkat and MCF-7, to examine whether this expression is cell-specific (Figure 3). Hsa_circ_HIPK3 expression was 2-fold downregulated in HeLa cells whereas its expression was upregulated in MCF7 and Jurkat cells 1.2 and 2-fold respectively. Expression of hsa_circ_0099768 was upregulated in HeLa and Jurkat cells. HeLa cells possessed the highest differential expression with a 6.7-fold change. Hsa_circ_0029693 was upregulated 5.6- and 2.1-fold in HeLa and MCF7 cells, respectively, while its expression was downregulated by 2.7-fold in Jurkat cells. All these results show that the induction of apoptosis results in differential expression of a number of circRNAs and their expression appears to be cell-specific at least for the candidate that we have analyzed in this study. Although our data provide an indirect relationship between the differential expression of certain circRNAs and apoptosis, further experiments are required to establish a direct link between an individual circRNA and apoptosis. For example, these candidates should be silenced or over-expressed to demonstrate the direct genotype-phenotype relationship.

**FIGURE 4:**
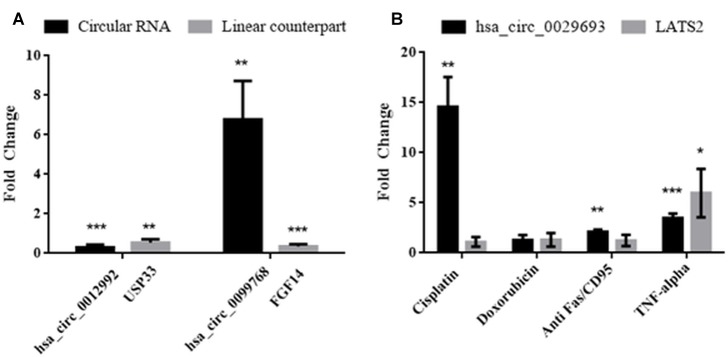
Analyses of expression levels of circular and their counterpart linear mRNAs. For circRNA analyses, experiments were carried out as explained in Figure 3. For mRNA analyses, RNAse R treatment was omitted before cDNA synthesis to amplify the linear RNAs. **(A)** HeLa cells treated with cisplatin only; **(B)** HeLa cells treated with cisplatin, doxorubicin, anti Fas, and TNF-alpha. Data, collected in three biological replicates, were normalized against DMSO control and GAPDH. ^∗^, ^∗∗^, and ^∗∗∗^ denote *p* ≤ 0.5, *p* ≤ 0.01, and *p* ≤ 0.001, respectively.

Molecular details of circRNA biogenesis are still in progress. It needs to be resolved whether canonical splicing precedes back-splicing or vice versa (exon skipping vs. direct back-splicing) (Chen and Yang, 2015). Recent evidence points to the use of constitutive exons in circRNA biogenesis at the expense of their linear counterpart (Aufiero et al., 2018). Thus, we subjected linear counterparts of the candidate circRNAs to qPCR analyses to examine whether circRNA expression is correlated with the template mRNA expression (Figure 4). LATS2, USP33, and FGF14 were the linear counterparts of hsa_circ_0029693, hsa_circ_0012992, and hsa_circ_0099768, respectively. In cisplatin-treated HeLa cells, USP33, the gene encoding a ubiquitin specific peptidase, was downregulated 2-fold in parallel with its circular counterpart hsa_circ_0012992, while hsa_circ_0099768 was upregulated 6.7-fold in apoptotic samples in contrary to its linear counterpart FGF14, the gene encoding fibroblast growth factor (Figure 4). The other candidate, has_circ_0029693 was also subjected to qPCR analysis with LATS2 in four drug/ligand-treated apoptotic samples. Both circular and linear counterparts were upregulated in TNF-alpha-induced apoptotic samples in 3.5- and 5.9-fold, respectively (Figure 4). These results suggest that has_circ_0099768 biogenesis is probably independent from its template mRNA biogenesis while the biogenesis of the other two candidates is correlated with the expression level of their template mRNAs. However, further analyses are required to examine the correlation between alternative splicing and circRNA biogenesis.

### CircRNA-miRNA Interactions and Coding Potential

One potential function of differentially expressed circRNAs is to serve as miRNA sponges to regulate the functions of miRNAs and their target mRNAs (Chen, 2016). To examine whether our circRNAs might regulate apoptosis through miRNA-circRNA interactions, we checked the potential miRNA binding sites on the candidate circRNAs. Origin mRNAs of candidate circular RNAs are obtained from CircInteractome tool by entering circular RNA ID. Potential miRNA binding sites were obtained by using starBase v3.0 which is an open-source platform for studying the miRNA-circRNA interactions from CLIP-seq (Li et al., 2014). To qualify as a miRNA sponge, a circRNA is expected to harbor multiple binding sites for the same miRNA as illustrated in the case of cdr1as-miR-7 interaction (Li et al., 2014; Chen, 2016).

**FIGURE 5:**
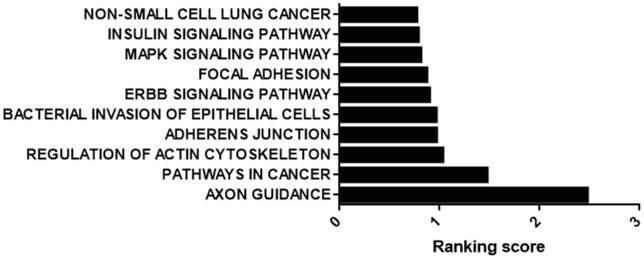
KEGG analysis of potential targets of miRNAs housed on candidate circRNAs. StarBase (starbase.sysu.edu.cn/) was used to identify potential miRNA-circRNA interactions in 109 differentially expressed circRNAs in the cisplatin-treated sample. miRNAs with five or more potential binding sites were filtered out and fed into miRSystem (mirsystem.cgm.ntu.edu.tw) to identify putative target mRNAs as well as pathways. The top 10 pathways were listed.

We prioritized the cisplatin-treated samples due to the cancer therapeutic potential of cisplatin. Interestingly, 71 out of 109 circRNAs possessed miRNA binding sites. Those 71 circRNAs were then classified based on the number of binding sites. miRNAs that were likely to bind to the same circRNA at least five times were analyzed by miRSystem. The target mRNAs of potentially sponged miRNAs were subjected to KEGG functional annotation analysis and the resulting top 10 pathways were presented in Figure 5. This analysis showed that the potential miRNA-circRNA interaction under our experimental setting is likely to modulate cellular processes such as pathways in cancer, cell adherence function and MAPK signaling pathway (Figure 5).

**FIGURE 6:**
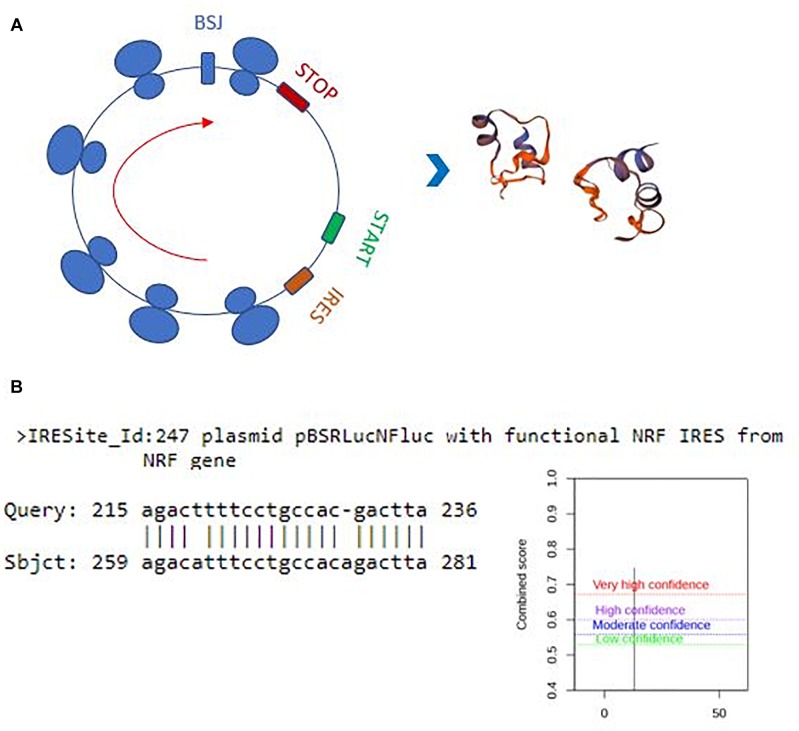
Translation potential of hsa_circ_0029693. Ensemble was used to identify the exon sources of circular candidates (ensembl.org/index.html). The circRNAs that originate from the first exons and harbor a potential internal ribosome entry site using IRESite (iresite.org/) were further examined by SRAMP (cuilab) for the presence of m^6^A sites. **(A)** Schematic representation of potential translation of hsa_circ_0029693 and 3D model of possible protein product. **(B)** The location of an IRES sequence and m^6^A methylation motif.

Perhaps, one of the most interesting functions of circRNAs is their translatability (Pamudurti et al., 2017). Potential translation of circRNAs could generate a truncated protein that could have a diverse array of functions in the cell. Thus, we also examined the translation potential of our candidates. Hsa_circ_0029693 originates from the 2^nd^ exon that includes half of the 5’UTR and the first translated exon of the linear source gene LATS2. The resulting circRNA is 444 nt that harbors a 147-aa open reading frame and an in-frame stop codon (Figure 6A). The 3D structure of putative truncated protein product of has_circ_0029693 was created by SWISS-MODEL ^7^. It might be a homodimer protein that contains caspase-3 and caspase-7 cleavage site motif but loses its protein kinase domain (Figure 6A). We used SRAMP (see text footnote 5) to check for potential m^6^A sites as these sites are potential indicator of translatability (Zhou et al., 2016). When we subjected has_circ_0029693 to such an analysis, a very high confident m^6^A site motif was detected in a site close proximity to translation start codon. Additionally, an IRES site was detected nearby the start codon (Figure 6B). All these analyses suggest that some of our candidates hold the potential for translation that could generate truncated products. Further experimental evidence would be required to conclusively demonstrate the translatability of circRNAs. For example, the polysome association of circRNAs would be a good support for their translatability. Perhaps, the most direct evident would be to detect these products (either the endogenous product or the over-expressed product) through mass spectroscopy. It will be quite interesting to investigate the potential role of these truncated products as they might potentially compete with the full- length proteins or have an entirely different function.

## Conclusion

Apoptosis is a highly regulated cellular process that is important for cell survival and cell death and it is involved in various physiological as well as pathological conditions. In this study, we provide a comprehensive profile of circRNAs in HeLa cells to another (Figure 1G). At least in HeLa cells, TNF-α and cisplatin are more potent in circRNA induction. However, doxorubicin and anti-Fas mAb treatment caused a moderate level of circRNA differential expression. It is difficult to account for this difference in the number of differentially expressed circRNAs. The consistent read number in all three biological replicates potentially point to a drug-specific response rather than a technical problem but this point requires further investigation. We then validated the existence of at least three circRNA candidates by PCR-amplification (Figure 2) and sequencing of the amplified fragments. qPCR analyses showed cell specific expression of circ-HIPK3 and hsa_circ_0029693 in HeLa, MCF-7 and Jurkat cells (Figure 3).

Interestingly, circRNAs, differentially expressed under apoptotic conditions, house binding sites for miRNAs reported to regulate apoptosis, suggesting a circRNA-miRNA-mRNA regulatory loop (Figure 5). Although we do not provide any experimental evidence at this point, our bioinformatics analyses provide an indirect link for a potential interaction between candidate circRNAs and miRNAs known to regulate apoptosis. However, more direct evidence would involve co-immunoprecipitation of circRNAs and miRNAs followed by PCR-amplification of the putative miRNAs. Also, silencing and/or over-expression would be helpful in demonstrating such direct interactions and their effect on the apoptotic phenotype. Our bioinformatics analyses also yielded interesting data at least with respect to the translatability of one candidate circRNA, has_circ_0029693. However, more solid evidence is required to claim the translation of a protein product from this circRNA.

In conclusion, although more experimental evidence is needed to demonstrate a direct link between differentially expressed candidate circRNAs and apoptosis, our data provide the first transcriptomics profile of circRNAs in HeLa cells. These data can be used in future studies to establish a direct link between candidates and apoptosis and also to study the effects of miRNA-circRNA interactions and putative truncated proteins on apoptosis.

## Data Availability

Raw sequence reads have been deposited in the GEO database (GSE125249).

## Author Contributions

BA obtained the funding and designed the experiments. BY and IE performed the experiments. BY performed bioinformatics analyses. BA, BY, and IE analyzed the data and wrote and approved the manuscript.

## Conflict of Interest Statement

The authors declare that the research was conducted in the absence of any commercial or financial relationships that could be construed as a potential conflict of interest.

## Footnotes

1 circinteractome.nia.nih.gov

2 ensembl.org/index.html

3 ncbi.nlm.nih.gov/orffinder/

4 cuilab.cn/sramp

5 starbase.sysu.edu.cn/

6 mirsystem.cgm.ntu.edu.tw

7 swissmodel.expasy.org
