# Supplementary material for: Transcriptomics Analysis of Circular RNAs Differentially Expressed in Apoptotic HeLa Cells
Source: Front Genet. 2019 Mar 13;10:176. doi: 10.3389/fgene.2019.00176 (PMC6424894; doi:10.3389/fgene.2019.00176)
Supplement: Supplementary file 1 [file Table_1.DOCX]

Supplementary Material

Transcriptomics analysis of circular RNAs differentially expressed in apoptotic HeLa cells

**Bilge Yaylak^1^, Ipek Erdogan^1^, Bunyamin Akgul^1*^**

*** Correspondence:** Corresponding Author: bunyaminakgul@iyte.edu.tr


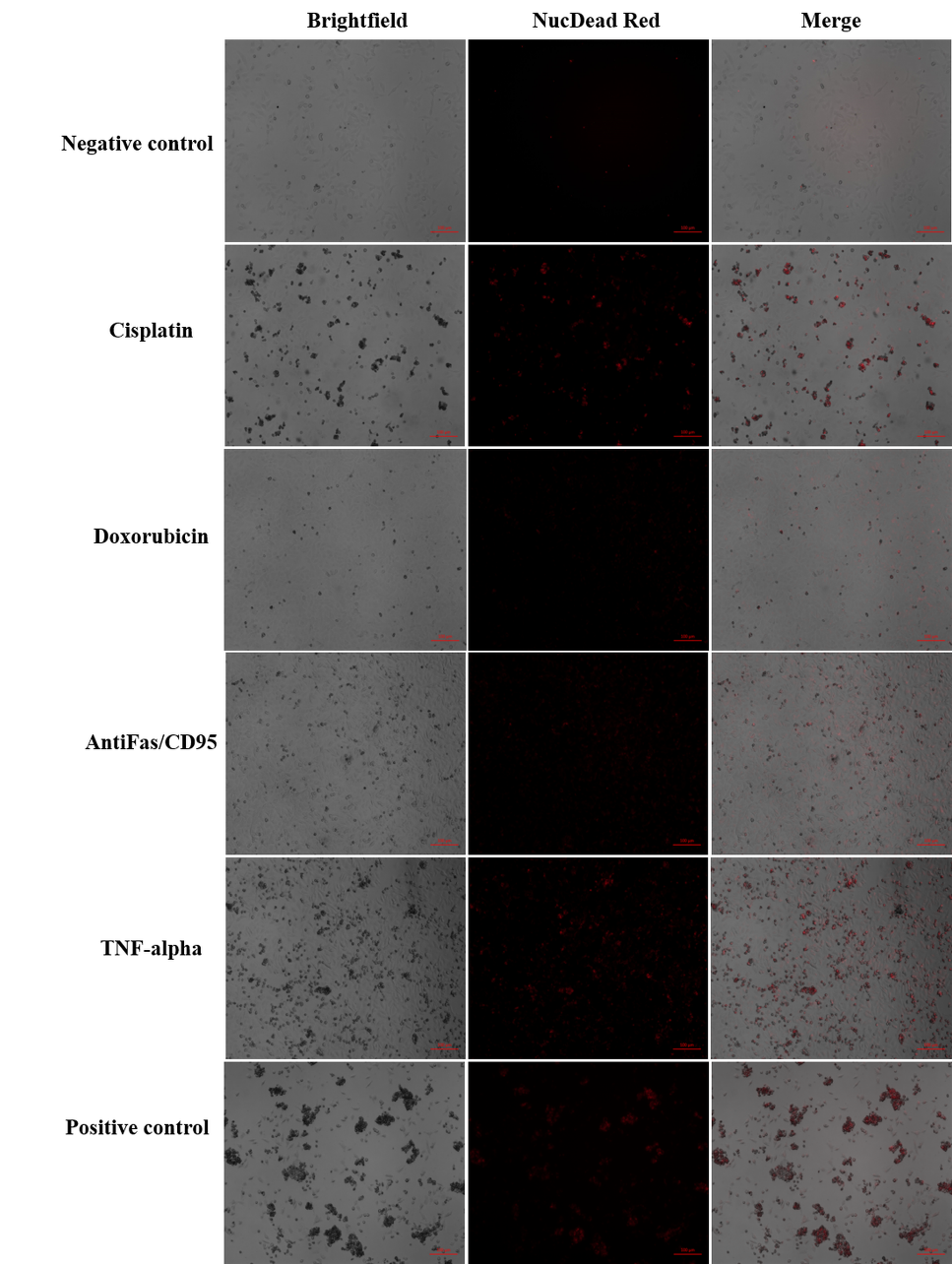


**Supplementary Figure 1. Micrographs of control and treatment groups of HeLa cells. Cells were stained with NucRed DeadTM 647 ReadyProbesTM Reagent after treatment with drugs/ligands.** Cells treated with 0.1% and 5% DMSO were used as negative and positive controls, respectively. Scale bar= 100 µm.


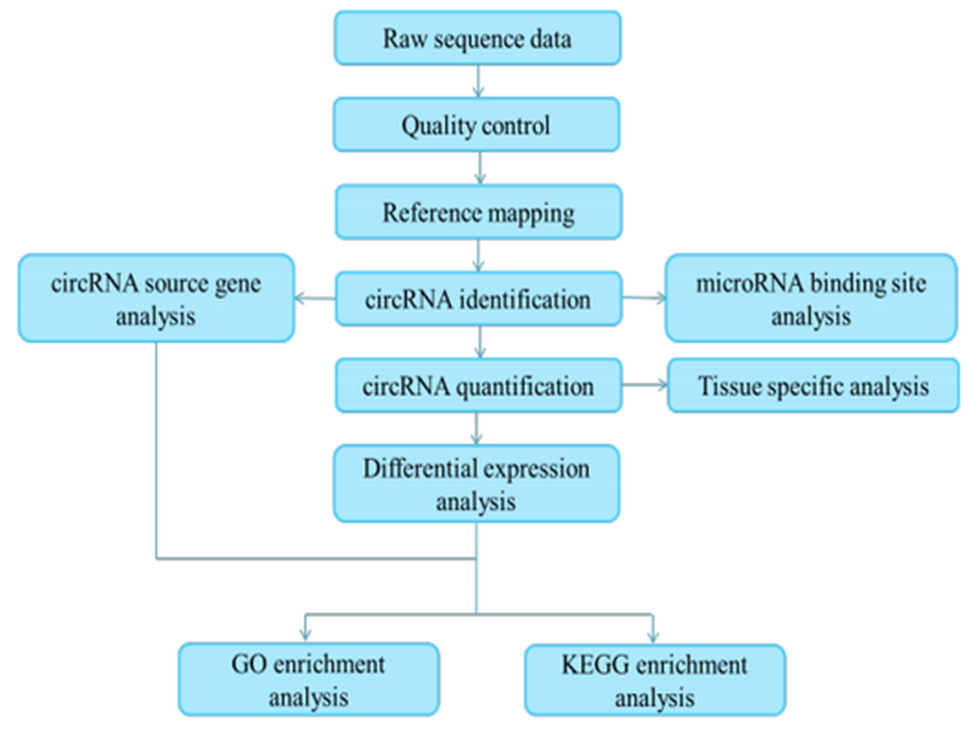


**Supplementary Figure 2. Flow chart of circRNA-seq analysis.**
